# Real-Time Performance of Mechatronic PZT Module Using Active Vibration Feedback Control

**DOI:** 10.3390/s16101577

**Published:** 2016-09-25

**Authors:** Francesco Aggogeri, Alberto Borboni, Angelo Merlo, Nicola Pellegrini, Raffaele Ricatto

**Affiliations:** 1 Department of Mechanical and Industrial Engineering, University of Brescia, via Branze, 38, 25123 Brescia, Italy; alberto.borboni@unibs.it (A.B.); nicola.pellegrini@unibs.it (N.P.); 2 CE.S.I Centro Studi Industriali, via Tintoretto, 10, 20093 Cologno Monzese, Italy; merlo@cesi.net; 3 FIDIA Spa, c.so Lombardia, 11, 10099 Torino, Italy; r.ricatto@fidia.it

**Keywords:** real-time control, mechatronics, PZT actuators, vibration, hardware in the loop

## Abstract

This paper proposes an innovative mechatronic piezo-actuated module to control vibrations in modern machine tools. Vibrations represent one of the main issues that seriously compromise the quality of the workpiece. The active vibration control (AVC) device is composed of a host part integrated with sensors and actuators synchronized by a regulator; it is able to make a self-assessment and adjust to alterations in the environment. In particular, an innovative smart actuator has been designed and developed to satisfy machining requirements during active vibration control. This study presents the mechatronic model based on the kinematic and dynamic analysis of the AVC device. To ensure a real time performance, a H2-LQG controller has been developed and validated by simulations involving a machine tool, PZT actuator and controller models. The Hardware in the Loop (HIL) architecture is adopted to control and attenuate the vibrations. A set of experimental tests has been performed to validate the AVC module on a commercial machine tool. The feasibility of the real time vibration damping is demonstrated and the simulation accuracy is evaluated.

## 1. Introduction

In modern machine tools, mechatronics may play a key role in improving machining performance and guaranteeing high quality and efficiency. Over the past years, a large amount of research has been developed to identify innovative solutions to minimize the undesirable effect of vibrations that seriously compromise the quality of the workpiece. This study aims at dealing with control and mitigation of vibrations in machining by proposing an innovative mechatronic device based on piezoelectric stack actuators. An active module is presented, composed of a host part integrated with sensors and actuators synchronized by a regulator able to make a self-assessment and adjust to the alterations in the environmental. In this way, mechanical dynamics and the control theories have been examined in designing smart piezoelectric structures.

The practice of piezoelectric materials as actuators of vibration control has been proved with success over the past twenty years [1,2]; Nevertheless, this field is still growing in terms of exploration and progress [1]. Vibrations represent one of the main issues that affect the quality of a workpiece, and they need to be considered from the design phase of a machine tool. They are usually classified in three main categories: free, self-induced, and forced vibrations. The first type of vibrations is related to pulsating excitations and it has a broad range of origins, such as imperfection of materials, inertia forces from mobile parts, or shocks transmitted by foundations. The second class of vibrations, known as chatter as well, is produced during the cutting process itself and they are induced by the unpredictability of the cutting phase. The last group of vibrations is generated by periodically varying forces due to bearing abnormalities, unstable effects, intermittent cutting, faulty gears, impact and motion of the foundations. There is a set of approaches able to control and mitigate vibrations. These methods focus on the design of the machine tool or on vibration compensation through passive and active control strategies. The design-based methods aim at making machine tools with structures that are stable, stiff, and able to dampen vibrations. State of the art offers a broad range of designs to reduce the weight of machine structures using innovative materials and achieving kinematic and dynamic performances [3,4,5]. Instead, the compensation-based methods include both traditional methods, called passive controls, and active control strategies. Passive controls aim at optimizing machine working parameters or using dampers and vibrations absorbers. They are not expensive, nevertheless, they may negatively impact machine efficiency and productivity. The alternative methods are the active controls which are based on structural systems that are able to alter machine dynamics by forming a closed loop through the installation of smart actuators and vibration sensors.

State of the art technology proposes a number of mechatronic applications on active vibration control [6]. Usually, these systems focus on smart bearings solutions, single degree of freedom piezo actuators, or voice coils applications [7]. Mechatronic products in the field of micro electro-mechanical systems (MEMS) are piezoelectric acceleration sensors, micro-actuators, and micro-pumps [8,9,10,11,12]. In particular, Shan et al. [13] presented two control techniques based on an axis frame mounted with a PZT transducer. Zhang et al. [14] conducted strain rate feedback control to suppress unwanted vibration of a manipulator with flexible parts. Flexible parallel manipulators are also used to achieve high structural vibration suppression using direct output feedback control [15].

**Figure 1:**
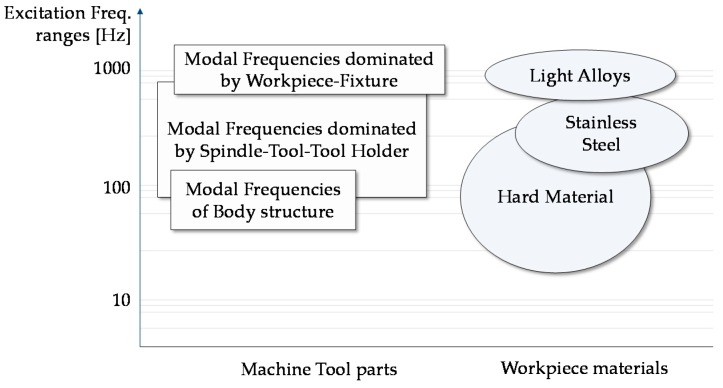
Summary of machine tool modal frequency ranges in turning and milling machining [21].

To identify the most suitable strategy, a study of vibration sources and frequencies is required. In machine tool analysis, the frequency modes are associated to the structural parts (commonly at low frequency) [16,17], tool, spindle system (influenced by rpm/speed) [18], and material workpiece [19,20]. Figure 1 summarizes the exciting frequency ranges of a set of materials in milling and turning machining. In general, hard material workpieces may generate vibrations with frequencies in a range below 200 Hz, while the frequency range defined from 160 Hz to 350 Hz is connected to machining of common steel materials. Light alloy materials have frequencies greater than 300 Hz. The undesirable effect of vibrations appears on the displacement of tool tip point which then generates irregularity, making the workpiece surface unacceptable for product quality.

This study presents an innovative active mechatronic module that aims at controlling and mitigating vibrations forced by a set of undesirable processes in the machine, such as unbalanced rotating masses, issues on gears, faulty bearing, and so forth. As shown above, the main sources of vibrations are connected to manufacturing operations and environmental conditions; an active control of vibration provides a number of advantages in avoiding potential breakdowns of machines by reducing vibrations, gaining highly efficient machining, and guaranteeing the required workpiece quality.

The proposed device has been designed with a focus on a light-weight structure and a compact design and is intended to be integrated into a micromilling machine. It has to link the electro-spindle with the vertical axis of a machine tool using 3 PZT actuators that permit the relative displacement of two platforms. This study covers the design and modeling of the mechatronic module. An experimental set-up on a commercial milling-machine tool has been performed to achieve the vibration damping and validate the active vibration control (AVC) module. The experimental outcomes are congruent with the Finite Element analysis and confirm the design assumptions.

## 2. Active Vibration Control (AVC) Module: Principles and Technical Features

**Figure 2:**
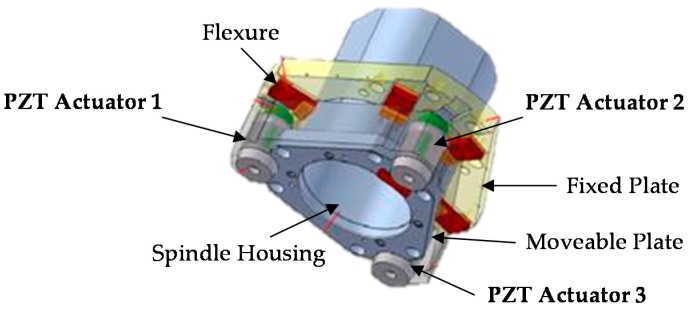
3D CAD Module overview.

**Figure 3:**
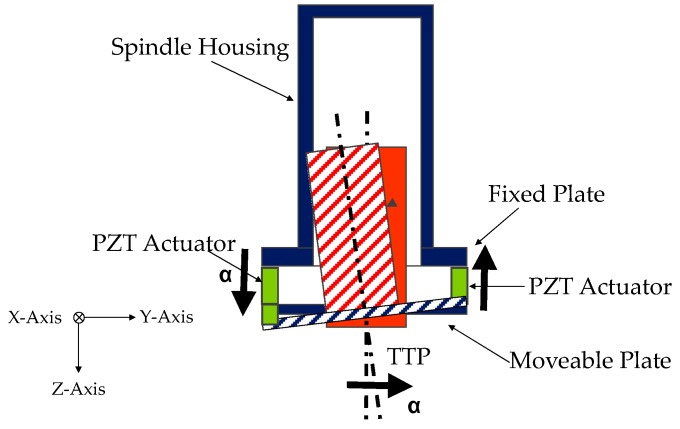
Module functional concept.

**Figure 4:**
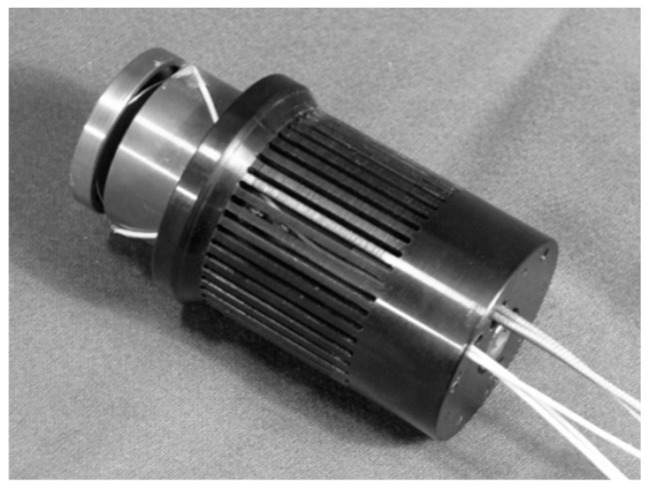
The smart actuator integrated into the mechatronic module.

This novel module is a smart element that deals with the vibration mitigation in micro-cutting processes. The main principle is to screen the vibratory frequency in order to control the displacement at the tool tip point of the machine. The proposed approach integrated mechatronic and control theories in the design phase. The device aims at increasing the quality of workpiece finishing through an AVC architecture based on high performance PZT-actuators. It is based on the Stewart platform [22,23,24,25,26], but it has only three degrees of freedom (DOFs) (two rotations on X, Y axes and one displacement on the Z axis). Figure 2 shows an overview of the AVC system that includes two platforms made of an Al alloy. The fixed platform is bound to the machine tool’s vertical axis, and the mobile is linked to the frame of the spindle. Three PZT multi-stack actuators permit the relative movement of these two platforms. The functional concept focuses on the recognition of undesirable displacements at the tool tip point. When a deviation is measured at the tool tip point (TTP), 3 actuators are switched on in order to smooth pulsations and limit their consequences on the surface, as shown in Figure 3. The piezo-electric element is able to manage compressive axial loads, but precautions are needed with tensile and/or shear forces; for this reason, special flexures have been designed and integrated to prevent any breakage or failure. These flexural joints are able to avoid torsional and shear stresses to the piezo elements. A mechanical system provides the suitable stiffness to the actuators, avoiding undesired stresses and breaks. In additions, two flexural springs are located close to every actuator. They have high torsional and radial stiffness, nevertheless, they are free to move in the axial direction. The module is also equipped with three steel C-shaped plates to increase the stiffness, located between the fixed platform and the actuators. Furthermore, every flexural joint is connected to a cooler joint that provides compressed air against the actuator surface when the temperature exceeds 40 °C. Figure 4 illustrates the smart actuator designed and developed to satisfy the machining requirements.

Considering the XYZ plane of Figure 3, Table 1 explains the motion strategy of the AVC device. An undesirable displacement on the X axis of the tool tip point is compensated for by a differential movement of the actuators. Under the small displacement hypothesis and supposing that the mobile plate of the platform is a rigid body, the kinematic correlation between the displacement at the tool tip point and actuation strokes is shown in Table 1.

In this way, the AVC module may be pointed out as a black block between two interfacings of the machine tool. The movement of the smart block is actuated by three piezo-electric stack actuators, which are located in a strategical position. The piezo electric actuation is regulated by the strains with sub-micron range sensitivity. An accelerometer converts the strokes at the TTP to be transformed into voltage of PZT-displacement. The module is also equipped by a set of sensors (force, temperature, and displacement) useful to permit and monitor the system functionalities. In particular, a temperature sensor is located on every actuator surface and a set of strain gauges is mounted on the steel disk. The main actuators’ technical features are listed in Table 2.

The proposed module needs to satisfy the Machine Tool life cycle time that is usually close to 30,000 h. In particular, the operative condition is the milling environment with temperature close to 40 °C, machining lubrication, and a frequency between 100 Hz and 300 Hz. The reliability analysis showed MTTF (Mean Time To Failure) of actuators close to 2.80E10 cycles (52,000 h at 150 Hz) satisfying the machine requirements.

This configuration may create some criticalities in terms of loss of stiffness (due to “in-series” arrangement of the piezo actuators). To avoid this problem, a set of parts (e.g., shaped plates, flexural springs) was added to systems to guarantee the required stiffness in XYZ directions. The FE analysis showed a reduction of spindle stiffness close to 6.9% in Z direction and 9.1% in X-Y directions. Nevertheless, this reduction does not impact on the required machining performance.

In order to identify the best location of the triaxial accelerometer to measure the tool tip point deviations, the dynamic stiffness of the mechatronic module was evaluated. In particular, the module architecture guarantees that the dynamic stiffness between the accelerometer point at the bottom ram flange and the tool tip is very high in frequency domain to assume that the reduction of vibration is the same for the two positions.

### 2.1. The Mechatronic Model

**Figure 5:**
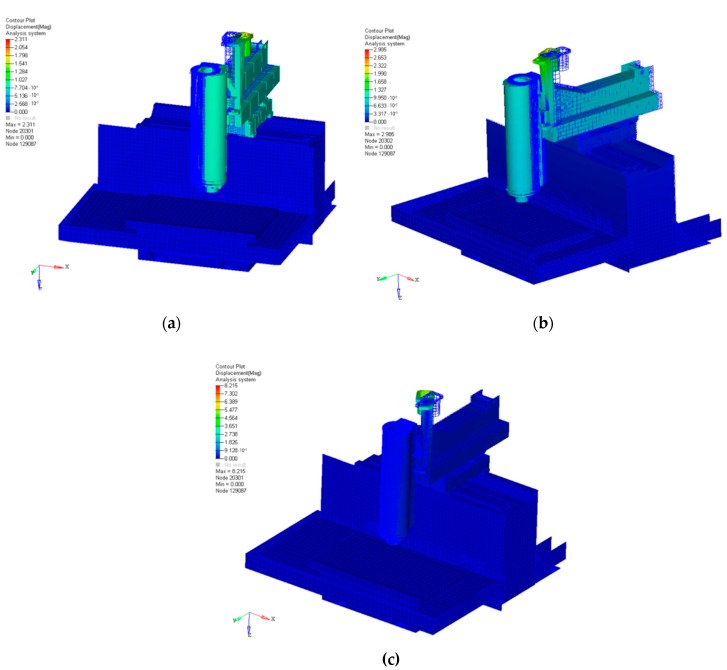
The FE modes 1–3 at 19 Hz (**a**); 24 Hz (**b**); and 30 Hz (**c**), respectively.

One of the most critical points in the control the AVC device is the identification of the correct mechatronic model; this includes a combination of multidisciplinary fields such as mechanical, electrical, control, and energy engineering. The first step is to evaluate the machine tool behavior using a set of simulations. In this way, a mechanical model may be represented by a Finite Element (FE) analysis. FE aims at describing the main characteristics of the machine tool, focusing on structural parts, links, and piezoelectric actuators (Figure 5). This study was developed on a commercial milling machine and the simulation results are presented in Table 3 and Figure 5. The FE model consists of approximately 150,000 elements.

To validate the simulation results, an experimental modal analysis was performed using the “hummer test”. Table 3 shows a comparison between the FE model frequencies and experimental data at different modes. The FE frequency modes are congruent with the experimental data, confirming the robustness of the numerical model. Figure 5 highlights a set of FE analysis examples.

In particular, FE analysis was replicated to evaluate the impact of the AVC device. It was noted that the mechatronic module introduced a frequency mode at 280 Hz, close to the critical frequency range for a standard milling operation. The aim of the control strategies was to suppress this mode and that of all others in order to improve the dynamic behavior of the machine tool.

The model was reduced in accordance to the Craig and Bampton technique [27,28,29] in order to facilitate dynamic simulations and testing. In this way, the degrees of freedom (DOFs) of any substructure were classified as boundary or internal. The basic assumption was that the sub-structure was characterized by defining the constrained modes and a few normal modes. In fact, it was useless to consider a large number of modes, as only a few of them had a physical meaning. Starting from the “FE model”, a “reduced FE” with a small number of DOFs have been identified.

In addition, the energy dispersion quantification of structural objects through vibration depended by several damping criteria. Damping parameters could not be quantified from similar structures or predicted by the Finite Element analysis.

In this study, the damping is assumed to be viscous and influenced by frequency. The assumption is to consider the damping condition as a linear formulation of mass and stiffness properties:

(1) C=α⋅[M]+β⋅[K]

where *α* and *β* are variables to be identified and [*M*] and [*K*] are the mass and stiffness matrices, respectively. The benefit of this construction is that the damping formulation is a diagonal matrix. The damping proportion *ξ* is associated to *α* and *β* through the following relation:

(2) $$\xi_{i}=\frac{\alpha }{2\cdot \omega_{i}}+\beta \cdot \frac{\omega_{i}}{2}$$

where *ω* is the Eigen-frequency of the i-th mode number. This method is applied to find damping characteristics within the normalized ranges, this is known as the half-power bandwidth technique. The results show that *α* varies from 0.05 to 0.10 while *β* is close to 9 × 10^−6^. In particular, it is noted that the mass is proportional to damping when α is greater than *β*.

Another critical point in defining the mechatronic model is the representation of the state space. This procedure aims at illustrating a system of n-first order differential equations. Therefore, the use of matrices simplifies the mathematical structure of the system equations. The growth of state variables, inputs, and outputs does not influence the complexity of these calculations. An AVC system and regulator need to work in real-time and for this reason the device behavior has to be explored in the time variable.

The state space (SS) model is derived from the corresponding FE model and it describes the dynamic behavior of the architecture using mathematical formulations. The dynamic scheme defines the coherence between the input and output parameters. Table 4 summarizes the SS model that has been created to control the device starting from the FE results.

In order to perform the simulation, the reduced FE model has been analyzed using Matlab-Simulink software. The reduced form is:

(3) $$\left\{M\right\}\cdot \ddot{z}+\left\{V\right\}\cdot z+\left\{K\right\}\cdot z=F$$

where *z* represents the path of movements of real DOFs, *F* is the force, {*M*} and {*K*} are the mass and stiffness matrices, respectively, and {*V*} is the damping matrix. Defined vector x(t), the matrices of state *A*, input *B*, and output *C* and *D* are deduced from the modal investigation. The dynamics of structure are defined by the SS form as follows:

(4) $$\begin{array}{l} x=\left\{\begin{array}{c} \dot{z}\\ z \end{array}\right\}\to \;\dot{x}=\left\{A\right\}\cdot x+\left\{B\right\}\cdot u\\ u=\left\{\begin{array}{c} F\\ 0 \end{array}\right\}\to \;y=\left\{C\right\}\cdot x+\left\{D\right\}\cdot u \end{array}$$

with

(5) $$A=\left\{\begin{array}{cc} -{\left[m\right]}^{-1}[v] & -{\left[m\right]}^{-1}[k]\\ \left[I\right] & \left[0\right] \end{array}\right\}$$

(6) $$B=\left\{\begin{array}{c} -{\left[M\right]}^{-1}\\ \left[0\right] \end{array}\right\}$$

{*C*} and {*D*} depend on selected variables, *x* is the state vector, and u represents the energy vector. The SS model of the AVC device may be created using FORTRAN code. This configuration is assimilated into SIMULINK to provide the dynamic reaction of the exhibited assembly (machine tool and AVC module) under defined perturbations.

### 2.2. Control Modeling and Strategies

The definition of the control strategy plays a key role in guaranteeing the AVC module functionality. State of the art technology presents a broad range of techniques to control AVC systems that may be classified in two main categories: active feedforward controllers and linear feedback regulators [30,31,32,33,34,35,36]. The first group of controllers provides stability and a straightforward physical implementation. Nevertheless, feedforward controllers may create a set of issues with a nonlinear feedback. The linear controllers are based on a regulator design and are able to manage any potential differentiation between the plant and the model [37].

A number of researches prefer to use linear representations for control strategy and simulation when micro-movements are examined [31,32,33,34,37]. The speed of the feedback architecture has a critical impact on the vibration dominance of smart assemblies.

In this study, a linear controller has been selected, in so far it is especially useful to control vibrations in flexible structures. As defined in the SS model, linear control methods have been considered using simulations to optimize the controller implemented in the real-time hardware. In order to decrease the vibration effect, a supervisor has been analyzed to manage the required signals (e.g., voltage). In this configuration, the perturbation signal of the TTP is an input of the regulator to be sent to the PZT-actuator in recovering the displacement. Vibration suppression has been evaluated using the multiple input - multiple output (MIMO) layout and the H2-Linear Quadratic Gaussian (LQG) regulator that together provide the chance to drive many actuators and manage huge sensor data [31,32] to reduce the white noise troubles.

Linear Quadratic Gaussian (LQG) optimal control is briefly introduced. An adjusted regulator factor may be achieved by reducing the following function:

(7) $$J_{LQG}=\int_{t_{0}}^{\infty }\left(y{\left(t\right)}^{T}\cdot \bar{Q}\cdot y(t)+u{\left(t\right)}^{T}\cdot \bar{R}\cdot u(t)\right)dt$$

where *Q* represents the power matrix of the device output and R is the device input matrix.

The Linear Quadratic Gaussian (LQG) method may be useful and effective in real manufacturing environments. In addition, the H2 formulation replaces the stochastic reading of the LQG technique using the down size of the two-norm of the closed-loop structure. This choice avoids the need to read factors such as the strength of the white noise that passes through the LQG control [36,37,38,39]. A closed loop control system needs to guarantee stability, performance, and robustness. These proprieties may be satisfied by evaluating the sensitivity function S and complementary sensitivity function T. In particular, the sensitivity S regulates the disturbance on the output of the control scheme while the complementary sensitivity T is significant for the closed-loop reaction and noise measurement. In particular, a robust control system design has to reduce S and T at low and high frequencies, respectively, and avoid vibration peaks.

In the light of these considerations, the selected regulator is based on LQG-H2 theory [32,33,34,35,36,37,38,39,40]. The main functional concept of the AVC device is to recognize any undesirable displacement on the tool tip point of the machine tool through a three-axial sensor. A regulator panel drives information from the accelerometer managing the dynamic signals to the three PZT actuators. The active structure is summarized as follows:

(8) $$\begin{array}{l} \dot{x}=\left\{A\right\}\cdot x+\left\{B_{1}\right\}\cdot w+\left\{B_{2}\right\}\cdot u\\ z=\left\{C_{1}\right\}\cdot x+\left\{D_{12}\right\}\cdot u\\ y=\left\{C_{2}\right\}\cdot x+\left\{D_{21}\right\}\cdot w \end{array}$$

Equation (8) show the SS calculation, where *x* is the state vector, *u* is the control vector, *w* is the input, *z* is the controlled variable (movement of TTP), and *y* is the recorded output. The H2 control needs a regulator based on the following transfer function:

(9) $$z(s)=G_{wz}(s)w(s)$$

such that ${\left\|G_{wz}\right\|}_{2}$ is negligible.

In this way, the H2 dynamic regulator assumes that:

(10) $$\begin{array}{l} \dot{\bar{x}}=(A-B_{2}\cdot K_{c}-C_{2}\cdot K_{e})\cdot \bar{x}+K_{e}\cdot y\\ u=-K_{c}\cdot \bar{x} \end{array}$$

where *Ke* and *Kc* are solutions of the Filter Algebraic Riccati Equation-Control Algebraic Riccati Equation [39]. The proposed solution is simple to manage, nevertheless, the assumptions restrict H2 optimization to the LQG framework.

### 2.3. Hardware In the Loop (HIL) Validation

In order to validate the mechatronic model, a set of simulations has been performed using Simulink software. The simulations involved the machine tool, PZT actuator, and controller models. In particular, the PZT actuator linear model has been included into the reduced machine tool representation.

**Figure 6:**
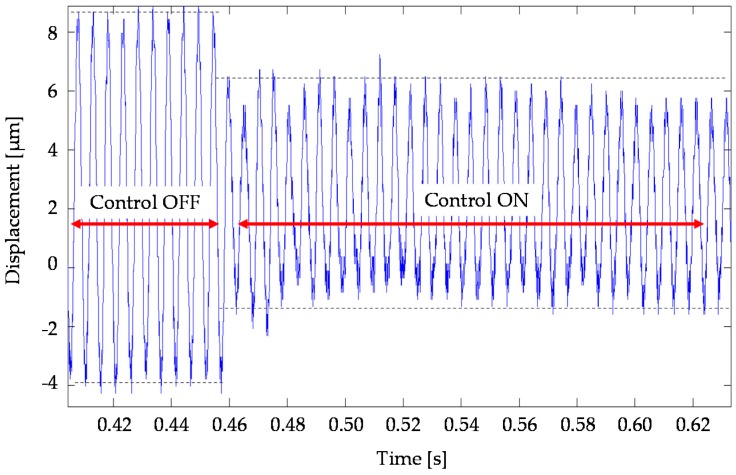
Simulation of activated/deactivate states of control of an unbalanced rotation at 11,500 rpm.

The main objective was to validate any different integrated model-testing control-schemes. A preliminary verification focused on a perturbation created by a digital input (sin-signal) to recreate the TTP displacement plotted in a time-domain. The simulation wanted to represent the undesirable, unbalanced tool rotation at 11,500 rpm. The control model was tested in both conditions (off/on) highlighting the effectiveness of the control system by 30–50% in terms of displacement amplitude reduction, as shown in Figure 6.

**Figure 7:**
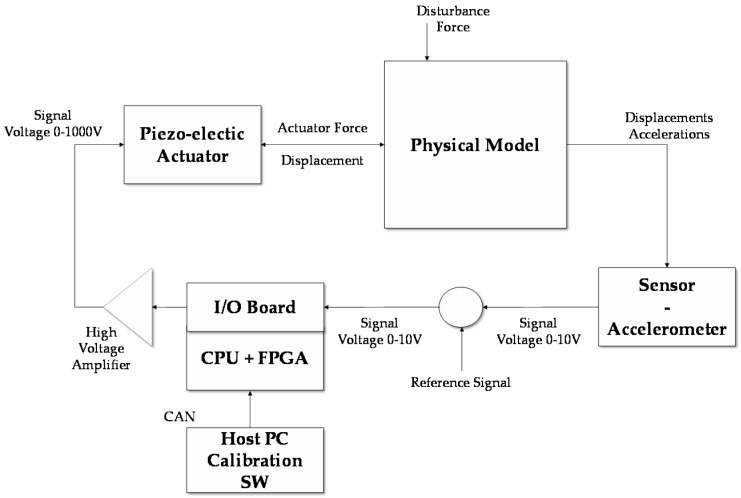
Hardware in the loop validation architecture.

To complete the preliminary validation of the regulator, a test bench was equipped with an electronic board consisting of a Matlab-based-FPGA (Field Programmable Gate Array) and a CPU (Central Processing Unit). The main advantage of applying a FPGA strategy is that the regulator is included as hardware, and it is very fast in comparison to microcontrollers. Figure 7 presents the block-wise flowchart of the regulator electronics with high-voltage amplifier. The regulator panel includes 12 bit A/D converters for analogue sensors (movement or acceleration) in addition to 16 bit D/A converters to create analogue signals for high-voltage power. The I/O (Input/Output) parts of the integrated circuit technology are conveyed on an I/O board and located in the CPU-FPGA board. The acceleration signal generates a differential control signal which is multiplied by different gains for the three different piezo-actuators in order to impose a displacement along the X direction. This scheme is simple in the parameter setting and easy to implement, nevertheless, it is strongly dependent on the dynamics of the active device (e.g., the sensor and the amplifier dynamics).

## 3. Experimental Tests and Results

A set of experimental tests was performed to validate the AVC system. As shown above, the control schemes were previously simulated by Simulink, then uploaded on the dSpace control personal computer. The main objective was to prove the feasibility of the control scheme using the acceleration as feedback. The choice to use the acceleration could complicate the evaluation of the feedback signal, but it was able to represent a broad range of applications. In this case, the displacement feedback could not be taken into consideration as it was a relative measurement.

**Figure 8:**
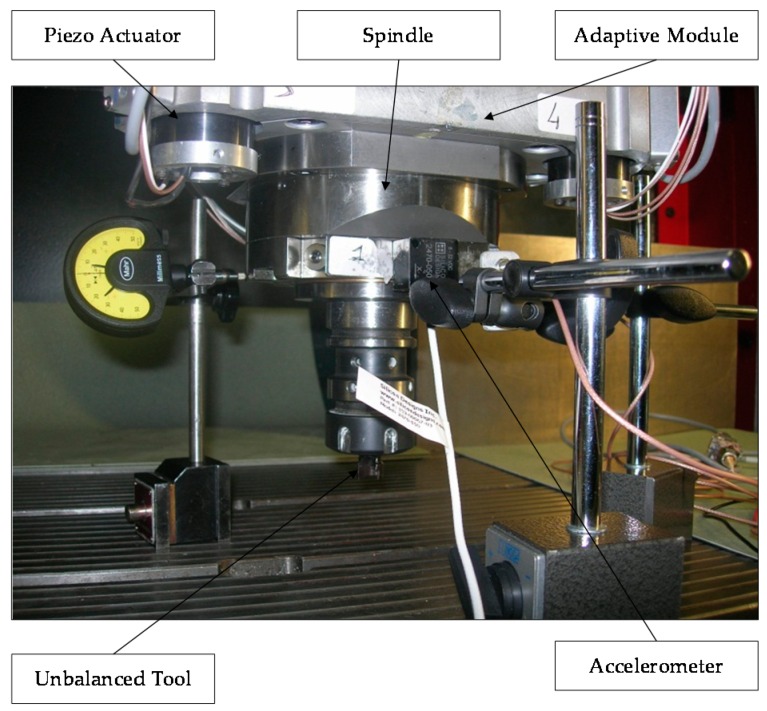
Experimental test overview.

In order to recreate undesirable conditions, the experimental tests were performed after unbalancing the tool. The aim of the tests was to validate the reduction of the TTP displacement due to the tool imbalance. A triaxial accelerometer was located close to the TTP to measure the vibration amplitude, Figure 8. In particular, the effect of the AVC system was analyzed in both conditions (off/on AVC module). The AVC device was tested considering a set of spindle frequencies, as follows: 10,500 rpm, 11,000 rpm, 11,200 rpm, and 11,500 rpm.

**Figure 9:**
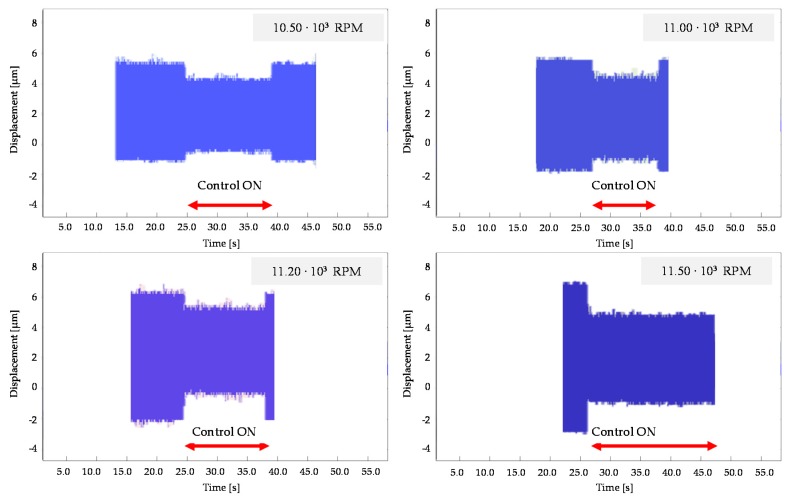
Experimental test of unbalanced spindle at different speeds considering on/off control.

Figure 9 presents the obtained results. For each spindle frequency, the effect of the AVC module is clearly visible in reducing the vibration impact.

**Figure 10:**
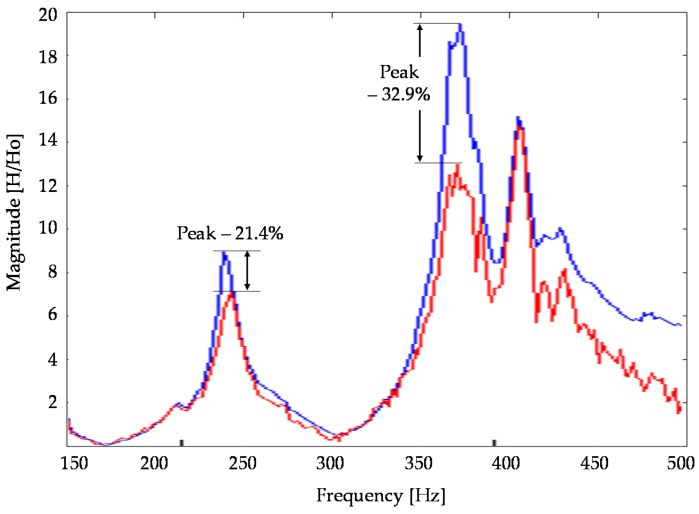
Frequency response function (FRF) of X axis with control (red line) and without control (blue line).

The improvement of the proposed device is also shown by the frequency response function (FRF), Figure 10. The vibration peaks between 200 Hz and 450 Hz are significantly reduced. Data over 450 Hz are not available due to the noise that occurred during the experimental tests. The results do not show particular benefits in reducing vibration at low frequency. This effect is mainly due to the accelerometer model and its sensitivity (0.1 V/m/s^2^). Future experimental tests will include a new MEMS accelerometer that is able to respond appropriately at a low frequency range.

**Figure 11:**
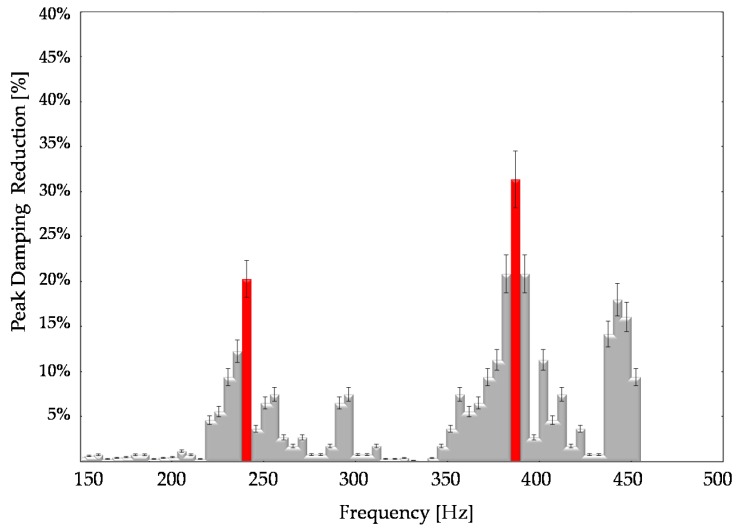
Residual vibration peak reduction percentage on X axis.

The FRF of Figure 11 underlines the comparison between the residual steady state of vibration amplitude and the uncontrolled states. The AVC module provides a suppression performance of 30%–35% at 380 Hz. Table 5 summarizes the main obtained results from experimental tests.

## 4. Discussion and Conclusions

This paper presents an AVC module to control and mitigate the effect of vibration in milling machining. In particular, a set of smart actuators has been designed and developed to satisfy machining requirements during active vibration control. A robust procedure was followed to study the mechatronic model, simulate the system behavior, and test the performance using experimental data. The AVC device is based on the measurement of the undesirable displacement at the tool tip point that activates three actuators that act to smooth pulsations and their consequences on the surface quality. To define the system architecture [39,41], the first step was to develop a FE analysis of the assembly (machine tool and AVC system) reduced by the Craig-Bampton technique. The mechatronic model focused on the definition of SS equations and the identification of the most suitable regulator. It was designed and optimized by using a linearized model. To ensure a real time performance, a H2-LQG controller was developed. In order to validate the mechatronic model, a set of simulations were performed using Simulink software. The simulations involved the machine tool, PZT actuator, and controller models. An algorithm was implemented on an integrated circuit board which receives data from the accelerometer and provides the required voltage to actuate PZTs in TTP displacement recovery. A set of experimental tests was executed to validate the AVC system using a commercial machine tool and a FPGA based controller. The experimental results show the AVC performance in reducing the displacement of the spindle TTP in the 250–400 Hz frequency range.

As shown in Table 6, state of the art technologies [41,42,43,44,45,46,47,48,49,50,51,52] highlight the potential benefits of the H2-LQG controller that was integrated in the proposed AVC device. In particular, the H2-LQG controller is one of the most promising regulators that provides an effective trade-off between displacement compensation and high accuracy performances in a broad frequency range (200–450 Hz). In the light of these considerations, the future research should investigate the effect of the MEMS accelerometer model on high frequency domains in order to extend this module architecture to a broad mechatronic sector. Further activities will be developed to reduce the AVC mass and improve the design compactness in order to increase its performance in vibration control.

## Figures and Tables

**Table 1:** Kinematic correlation between displacement at the tool tip point and actuation strokes.

Tool Tip Point (TTP)	Actuation Strokes
Δ TTP (X; Y; Z) = (1; 0; 0)	Act (Act1; Act2; Ac3) = (+1.0; 0.0; ‒1.0)
Δ TTP (X; Y; Z) = (0; 1; 0)	Act (Act1; Act2; Ac3) = (‒0.5; 1.0; ‒0.5)
Δ TTP (X; Y; Z) = (0; 0; 1)	Act (Act1; Act2; Ac3) = (+1.0; 1.0; +1.0)

**Table 2:** Actuators’ technical features.

Technical Features	Value
Length	60 mm
El capacitance	800 nF
Stiffness	450 N/μm
Resonance Frequency	30 kHz
Maximum Load	35 kN
Maximum Force Generation	25 kN
Maximum Tensile Force	4 kN

**Table 3:** Comparison between experimental and numerical mode and frequencies. FE stands for Finite Element.

Mode	FE Model Freq (Hz)	Experimental Freq (Hz)	Damping
1	19	21.6	0.17
2	24	24.3	0.09
3	30	34.8	0.04
6	53	49.1	0.03
7	61	59.3	0.02
8	71	68.2	0.05
10	80	84.1	0.04

**Table 4:** State space model variables.

Inputs	Outputs
Forces on the TTP on X, Y, and Z axes	Elongation of the piezo actuators (strain measure)
Forces acting on the piezo actuators	Distance between moveable module and fixed plate on three points (located on piezo actuators)
Forces acting on the kinematic chains X and Y	Accelerations (X, Y, Z axes) measured
	Displacement of TTP (X, Y, Z axes) elongation of the kinematic chains

**Table 5:** Experimental real time effectiveness.

Frequency Range (Hz)	Control OFF (Peak Magnitude)	Control ON (Peak Magnitude)	Peak Reduction (%)
230–240	8.92	7.01	21.4%
370–380	19.36	12.98	32.9%

**Table 6:** Trade-off comparison of different control approaches (advantage (+)/disadvantage (–)).

	Robust Control		Adaptive Control		Intelligent Control	
Disturbance source	H2-LQG Proposed		Model Reference Adaptive Control (MRAC)	Dual Control	Neural Networks Control (NNC)	Fuzzy Logic Control (FLC)
Machining Parameters (Axis position, Spindle RPM, Feed rate, etc.)	(+)	Easy to implement	Negligible response on the system	Low time to reach convergence, process parameters variation is rapid	Simple programming	Based on expert knowledge
	(−)	One operative range	Difficult to develop	Suboptimal solution needed	Convergence is time consuming	Difficult for MIMO system without adaption
Actuation Parameter Characteristics	(+)	Easy to implement	Negligible response on the system	Process parameters variation is rapid	Simple programming	Extremely simple to implement
	(−)	One operative range	Convergence is time-consuming	Extremely difficult to implement	Many data to be fitted	Difficult for MIMO system without adaption
Missing Information after FE Model Reduction	(+)	Easy to implement	Negligible response on the system	Low time to reach convergence	Best model uncertainties, simple programming	Based on expert knowledge
	(−)	One operative range	Convergence is time-consuming	Extremely difficult to implement, suboptimal solution needed	Many data to be fitted	Difficult for MIMO (Multiple Input Multiple Output) system without adaption

## References

[1] Vepa R. (2010). Dynamics of Smart Structures.

[2] Piefort V. (2001). Finite Element Modelling of Piezoelectric Active Structures. Ph.D Thesis.

[3] Ashby M.F., Evans A.G., Fleck N.A., Gibson L.J., Hutchinson J.W., Wadley H.N.G. (2000). A Metal Foams, Design Guide.

[4] Archenti A., Nicolescu C.M. Model-based Identification of Dynamic Stability of Machining System. Proceedings of the 1st International Conference on Process Machine Interaction.

[5] Schmitz T.L., Ziegert J.C., Canning J.S., Zapata R. (2008). Case study: A comparison of error sources in high-speed milling. Precis. Eng..

[6] Denkena B., Möhring H.-C., Will J.C., Sellmeier V. (2006). Stability considerations of a piezo-electric adaptronic spindle. Wt-Online.

[7] Kern S., Roth M., Abele E., Nordmann R. (2006). Active Damping of Chatter Vibrations in High Speed Milling Using an Integrated Active Magnetic Bearing.

[8] Gad-el-Hak M. (2000). MEMS Handbook.

[9] Madon M. (2001). Fundamentals of Microfabrication.

[10] Lyshevski S.E. (2001). Nano- and Micro-Electro-Mechanical Systems.

[11] Janocha H. (2004). Actuators, Basics and Principles.

[12] Slatter R., Degen R. Micro actuators for precise positioning applications in vacuum. Proceedings of the 9th International Conference on New Actuators, Actuator 2004.

[13] Shan J., Liu H.T., Sun D. (2005). Slewing and vibration control of a single-link flexible manipulator by positive position feedback (PPF). Mechatronics.

[14] Zhang X., Mills J.K., Cleghorn W.L. (2009). Flexible linkage structural vibration control on a 3-PRR planar parallel manipulator: Experimental results. J. Syst. Control Eng..

[15] Zhang Q., Mills K.J., Cleghorn L.W. (2013). Trajectory tracking and vibration suppression of a 3-PRR parallel manipulator with flexible links. Multibody Syst. Dyn..

[16] Altintas Y., Brecher C., Weck M., Witt S. (2005). Virtual machine tools. CIRP Ann. Manuf. Technol..

[17] Catania G., Mancinelli N. (2011). Theoretical–experimental modeling of milling machines for the prediction of chatter vibration. Int. J. Mach. Tools Manuf..

[18] Cao Y., Altintas Y. (2007). Modelling of spindle-bearing and machine tool systems for virtual simulation of milling operations. Int. J. Mach. Tools Manuf..

[19] Bravo U., Altuzarra O., Lopez de Lacalle L.N. (2005). Stability limits of milling considering the flexibility of the workpiece and the machine. Int. J. Mach. Tools Manuf..

[20] Campa F.J., Lopez de Lacalle L.N., Celaya A. (2010). Chatter avoidance in the milling of thin floors with bull-nose end mills: Model and stability diagrams. J. Mach. Tools Manuf..

[21] Dequidt A., Castelain J.M., Valdes E. (2000). Mechanical pre-design of high performance motion servomechanisms. Mech. Mach. Theory.

[22] Dietmair A., Zulaika J.J., Sulitka M., Bustillo A., Verl A. Lifecycle impact reduction and energy savings through lightweight eco-design of machine tools. Proceedings of the 17th CIRP International Conference on LCE.

[23] Zulaika J.J., Campa F.J., Lopez de Lacalle L.N. (2011). An integrated process–machine approach for designing productive and lightweight milling machines. Int. J. Mach. Tools Manuf..

[24] Altintas Y., Woronko A. (2002). A Piezo Tool Actuator for precision turning of hardened shafts. CIRP Ann. Manuf. Technol..

[25] Denkena B., Gummer O., Will J.C., Hackelooer F. Compensation of static and dynamic tool deflections during milling processes by an adaptronic spindle system. Proceedings of the 2nd International Conference on Innovative Cutting Processes & Smart Machining.

[26] Drossel W.G., Wittstock V. (2008). Active spindle support for improving machining operations. CIRP Ann. Manuf. Technol..

[27] Radecki P., Kruse W., Welsh A., Moro E., Park G., Bement M. Improving a turning process using piezoelectric actuators. Proceedings of the IMAC-XXVII.

[28] Abi Hanieh I., Preumont A., Loix N. Piezoelectric stewart platform for general purpose active damping interface and precision control. Proceedings of the European Space Mechanisms and Tribology Symposium.

[29] Craig R.R., Bampton M.C.C. (1968). The coupling of substructures for dynamic. AAIA.

[30] Ghareeb N., Weichert D. Combined multi-body-system and finite element analysis of a complex mechanism. Proceedings of the 11th SAMCEF User Conference.

[31] Fan J.P., Tang C.Y., Chow C.L. (2004). A multilevel superelement technique for damage analysis. Int. J. Damage Mech..

[32] Hughes P.C. (1987). Space structures vibration modes: How many exist which ones are important. IEEE Control Syst. Mag..

[33] Raja S., Sinha P.K., Prathap G., Bhattacharya P. (2002). Influence of one and two dimensional piezoelectric actuation on active vibration control of smart panels. Aerosp. Sci. Technol..

[34] Gawronski W.K. (2004). Advanced Structural Dynamics and Active Control of Structures.

[35] Li F.M., Song Z.G., Chen Z.B. (2012). Active vibration control of conical shells using piezo-electric materials. J. Vib. Control.

[36] Zhou K., Doyle J.C. (1998). Essentials of Robust Control.

[37] Robl C., Englberger G., Farber G. H2-Control with acceleration feedback for a micro positioning system. Proceedings of the 1999 IEEE International Conference on Control Applications.

[38] Lin J.C., Nien M.H. (2005). Active control of a composite cantilever beam with piezo electric damping-modal actuators/sensors. Compos. Struct..

[39] Zapateiro M., Karimi H.R., Luo N., Phillips B.M., Spencer B.F. A mixed H2/HN-based semiactive control for vibration mitigation in flexible structures. Proceedings of the 48th IEEE Conference on Decision and Control and 28th Chinese Control Conference.

[40] Hu Y.R., Vukovich G. (2005). Active r obust shape control of flexible structures. Mechatronics.

[41] Aggogeri F., Al-Bender F., Brunner B., Elsaid M., Mazzola M., Merlo A., Ricciardi D., de la O Rodriguez M., Salvi E. (2013). Design of piezo-based AVC system for machine tool applications. Mech. Syst. Signal Process..

[42] Foutsitzi G., Marinova D.G., Hadjigeorgiou E., Stavroulakis G.E. Robust H2 vibration control of beams with piezoelectric sensors and actuators. Proceedings of the 2003 International Conference on Physics and Control.

[43] Aguirre G., Al-Bender F., Van Brussel H. (2010). A multiphysics model for optimizing the design of active aerostatic thrust bearings. Precis. Eng..

[44] Bevly D., Dubowsky S., Mavroidis C. (2000). A Simplified Cartesian-Computed Torque Controller for Highly Geared Systems and its Application to an Experimental Climbing Robot. MIT J. Dyn. Syst. Meas. Control.

[45] Holterman J., De Vries T. (2005). Active Damping Based on Decoupled Collocated Control. IEEE/ASME Trans. Mech..

[46] Kandhil T.H. (2005). Adaptive feedforward cancellation of sinusoidal disturbances in superconducting RF cavities. Nuclear Instrum. Method Phys. Res..

[47] Nguyen-Tuong D., Peters J. Learning Robot Dynamics for Computed Torque Control Using Local Gaussian Processes Regression. Proceedings of the ECSIS Symposium on Learning and Adaptive Behaviors for Robotic System.

[48] Preumont A., Dufour J.P., Malékian C. (1992). Active Damping by a Local Force Feedback with Piezoelectric Actuators. AIAA J. Guid. Control Dyn..

[49] Symens W., Van Brussel H., Swevers J. (2004). Gain-scheduling Control of Machine Tools with Varying Structural Flexibility. Ann. CIRP.

[50] Tjahjowidodo T., Al-Bender F., Van Brussel H., Symens W. (2007). Friction characterization and compensation in electro-mechanical systems. J. Sound Vib..

[51] Van Brussel H.M.J. (1996). Mechatronics—A Powerful Concurrent Engineering Framework. IEEE/ASME Trans. Mech..

[52] Mazzola M., Aggogeri F., Merlo A., Brunner B., Rodriguez M., De La O. Reliability characterization of a piezoelectric actuator based AVC system. Proceedings of the 10th ASME Biennial Conference on Engineering Systems Design and Analysis.

